# Anthropogenic climate change is causing stronger and more extreme western Pacific subtropical high

**DOI:** 10.1093/nsr/nwag497

**Published:** 2026-08-17

**Authors:** Yanxin Zheng, Jeremy Cheuk-Hin Leung, Shuanglin Li, Banglin Zhang

**Affiliations:** Heavy Rain and Drought-Flood Disasters in Plateau and Basin Key Laboratory of Sichuan Province, Institute of Tibetan Plateau Meteorology, China Meteorological Administration, Chengdu 610213, China; Institute of Tibetan Plateau Meteorology, Chinese Academy of Meteorological Sciences, Beijing 100081, China; Department of Atmospheric Science, China University of Geosciences, Wuhan 430074, China; College of Meteorology and Oceanography, National University of Defense Technology, Changsha 410073, China; Laboratory of Atmospheric Environmental Monitoring and Early Warning for Low-Altitude Economy, National University of Defense Technology, Changsha 410073, China; Department of Atmospheric Science, China University of Geosciences, Wuhan 430074, China; Nansen-Zhu International Research Centre, Institute of Atmospheric Physics, Chinese Academy of Sciences, Beijing 100029, China; College of Meteorology and Oceanography, National University of Defense Technology, Changsha 410073, China; Laboratory of Atmospheric Environmental Monitoring and Early Warning for Low-Altitude Economy, National University of Defense Technology, Changsha 410073, China; College of Atmospheric Science, Lanzhou University, Lanzhou 730000, China; Key Laboratory of High Impact Weather (Special), China Meteorological Administration, Changsha 410073, China

**Keywords:** western Pacific subtropical high, anthropogenic climate change, thermodynamic-dynamic synergy, joint probability distribution frequency, extreme weather

## Abstract

The western Pacific subtropical high (WPSH) is a crucial system controlling weather anomalies in East Asia. Early studies defined the WPSH using either thermodynamic or dynamic metrics, but these metrics yield inconsistent trends and coverages in WPSH under anthropogenic warming. Subsequently, consensus on the response of WPSH to climate change has not been reached. Here, we investigate the response of WPSH to past and future climate change by developing a novel WPSH definition based on thermodynamic-dynamic synergy, which provides higher trend consistency of WPSH and better spatiotemporal agreement with weather anomalies. Our results reveal robust increases in WPSH area (0.25–1.03 × 10^5^ km^2^/decade) and intensity (0.35–1.98 × 10^5^ km^2^ gpm/decade), as well as a northwestward extension of WPSH boundary (−0.18 to −0.32 °E/decade), under a warmer climate, which are consistent across datasets and supported by a strong inter-model consensus. Particularly, climate models project an increased likelihood of record-breaking WPSH events on both seasonal and synoptic scales, with stronger intensity, longer durations, and higher occurrences under higher-emission scenarios. The findings indicate an increasing exposure of human societies to intense monsoon rainfall and heatwave risks associated with WPSH beyond historical records.

The western Pacific subtropical high (WPSH) is a vital component of the East Asian summer monsoon system. Variations in its intensity, coverage, as well as shifts in its positioning, are key factors leading to weather and climate anomalies. In recent years, an increasing frequency of extreme droughts and floods in East Asia during summer has been observed, with their intensity and duration constantly surpassing historical records, which can be attributed to the anomalous activities via the horizontal and vertical airflows associated with the WPSH [1–3]. Therefore, it is crucial to understand how the WPSH responds to recent and future anthropogenic climate change.

The WPSH has been reported to exhibit frequent abnormal westward extension under a warmer climate, crossing the Tibetan Plateau and connecting with the Iranian High, which exerts profound impacts on weather and climate [4,5]. However, the above conclusions may be questionable because the validity of WPSH definitions under climate change has seldom been accounted [6–8]. Namely, at present, the fixed geopotential height contour at 500 hPa (S-5875 hereafter, see ‘WPSH Indices’ in ‘Methods,’ Supplementary Data) is the commonly used definition of WPSH boundary by meteorologists from China, Korea, and Japan [1,9]. This is because the western flank of WPSH marks the boundary between moist and warm conditions, and its westward extension corresponds to a westward shift of the rainband, accompanied by an expansion of the subsiding warm and dry region within the WPSH. However, greenhouse warming has led to a thickening of the troposphere (Fig. 1d, black contour), hence an increase in the overall atmospheric geopotential. In such sense, the increase and westward expansion of the WPSH boundary in S-5875 (Fig. 1c) could be the result of the warming-induced thickening of the troposphere, and should not be considered as direct evidence of the strengthening WPSH [10,11].

**Figure 1.**
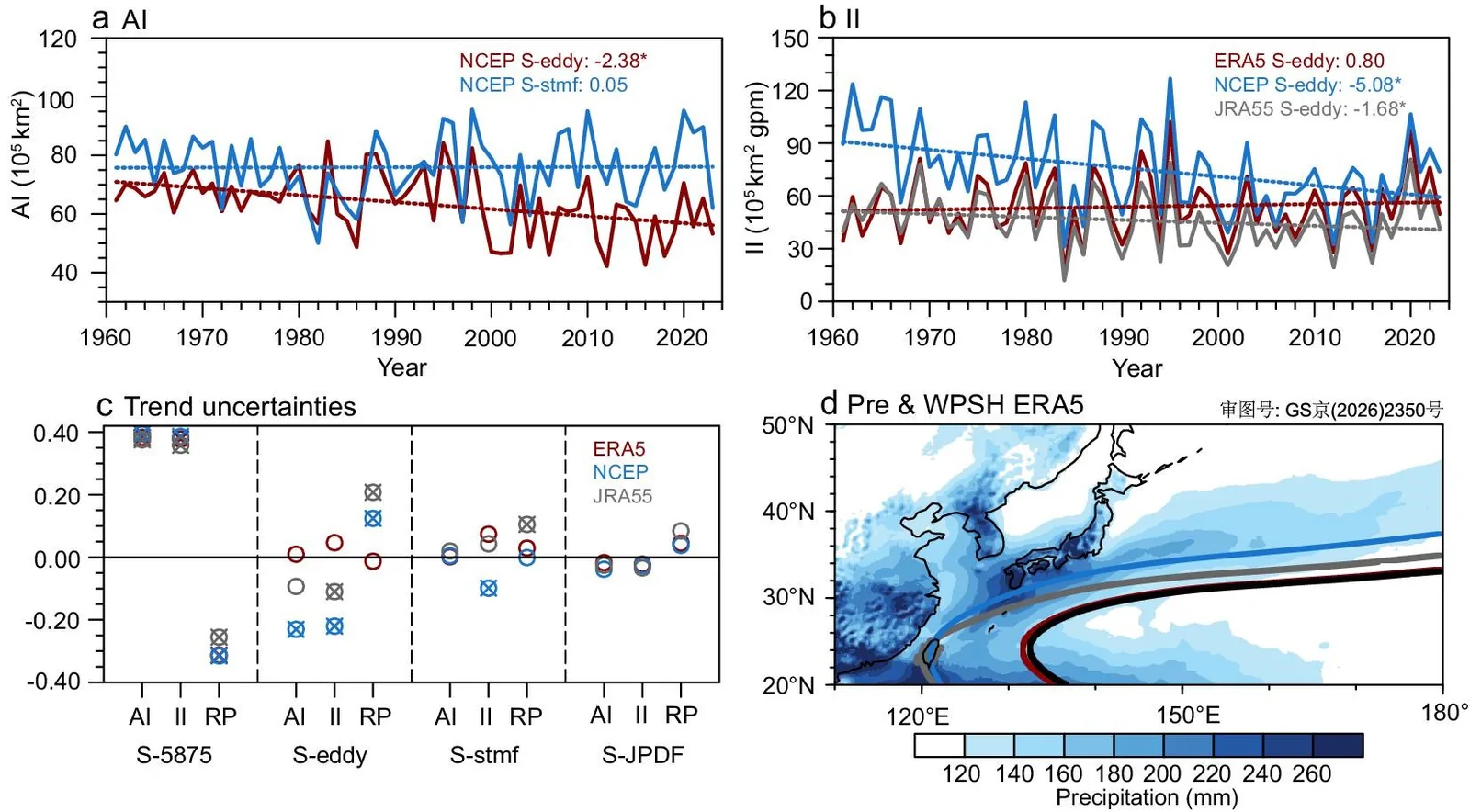
(a) The evolution and trend of the AI index (units: 10^5^ km^2^) in different definitions in the NCEP1 dataset. (b) The evolution and trend of the II index (units: 10^5^ km^2^ gpm) in S-eddy definition across different datasets. The values above each panel show the trend (per decade). The asterisk denotes the trend is statistically significant at the 99% confidence level. (c) Trends of normalized WPSH AI (Area index), II (Intensity index), and RP (Western Ridge Point index) derived from different WPSH definitions and three reanalysis datasets in 1961–2023. Circles with crosses denote trends significant at the 99% confidence level. (d) The summer boundary lines of the WPSH against the precipitation distribution in 1961–2023 in ERA5. The black, gray, blue, and red contours represent the results in S-5875, S-eddy, S-stmf, and S-JPDF, respectively.

In order to address this issue, several WPSH metrics have been designed to study the long-term changes in the WPSH, based on either thermodynamic or dynamic metrics [12,13]. However, the changes in WPSH estimated from different definitions show disagreements with each other [8,14]. The thermodynamic-based WPSH metrics, represented here by the eddy geopotential height definition (S-eddy hereafter, see ‘Methods’), indicate that the WPSH has weakened and retreated eastward during the past decades, which could explain the northward shift and eastward retreat in typhoon tracks, and the trend will continue in the future climate [15,16]. In contrast, the dynamic-based WPSH metrics, exemplified here by the streamfunction definition (S-stmf hereafter, see ‘Methods’), exhibit no obvious trend in the past decades, although the strength of the WPSH will increase in the future [14,17]. This conclusion is manifested by the enhancement of the summer monsoon circulation and increases of moisture transport farther into the inner Asian continent [18]. More importantly, a large inter-model spread is observed in both dynamic- and thermodynamic-based WPSH metrics. About half of the models project an enhanced WPSH, while the other half indicate a weakening in various emission scenarios [19–21]. Here we reproduced the above results in prior publications and verified the definition dependence of the WPSH (see ‘Disagreements in WPSH’ in ‘Methods,’ Figs 1 and S1). These uncertainties in the WPSH changes among the definitions arise because these metrics treat the thermodynamic and dynamic features of WPSH separately. While the thermodynamic-based metrics regard WPSH as a warm high-pressure system, the dynamic-based metrics regard the WPSH as a deep anticyclonic circulation. The differing response of the thermodynamic and dynamic features to warming may cause uncertainties for WPSH-related research on climate change [16,22].

Given that the WPSH is constrained by dynamic and thermodynamic features, this work innovatively proposes a concise yet comprehensive WPSH definition that integrates thermodynamic-dynamic synergies. It resolves the uncertainty caused by one-sided definitional metrics and provides a more reliable characterization of WPSH change under a warming climate. Based on the new definition, we find that the WPSH exhibits a robust enhancement in area and intensity with a strong inter-model consensus, and that record-breaking WPSH events will become more frequent on both seasonal and synoptic scales in a warmer climate, thereby elevating the risk of extreme floods and heatwave disasters with profound societal and economic consequences.

## RESULTS

### WPSH based on the S-JPDF

To capture the thermodynamic-dynamic synergy of the WPSH, we utilize the joint probability distribution frequency (JPDF) between 500-hPa geopotential height (Z) and vertical velocity (ω) activity over East Asia. The motivation for employing Z and ω to identify the WPSH is detailed in the Method-JPDF section. The Z-ω JPDF determines the spatial probability at which (i.e., the area of grids in which) certain values of ω and Z appear in a field. The climatological Z-ω JPDF over 1961–2023 reveals that as the Z values increase, the overall ω increases. That is, larger geopotential is accompanied by stronger downward airflow and a reduction in ω variance (Fig. 2a). Over the past 63 years, the climatological Z-ω JPDF exhibits an overall rightward-shifting trend, with decreases/increases in the probability of low/high Z values. This is because warming has led to an overall thickening of the air column according to the hydrostatic equilibrium equation (whereby air volume increases with the rising temperature), while no significant change is observed in vertical airflow. Similar Z-ω JPDF patterns are obtained across different reanalyses (Fig. S2c, d), with slight differences that may arise from different resolutions.

**Figure 2.**
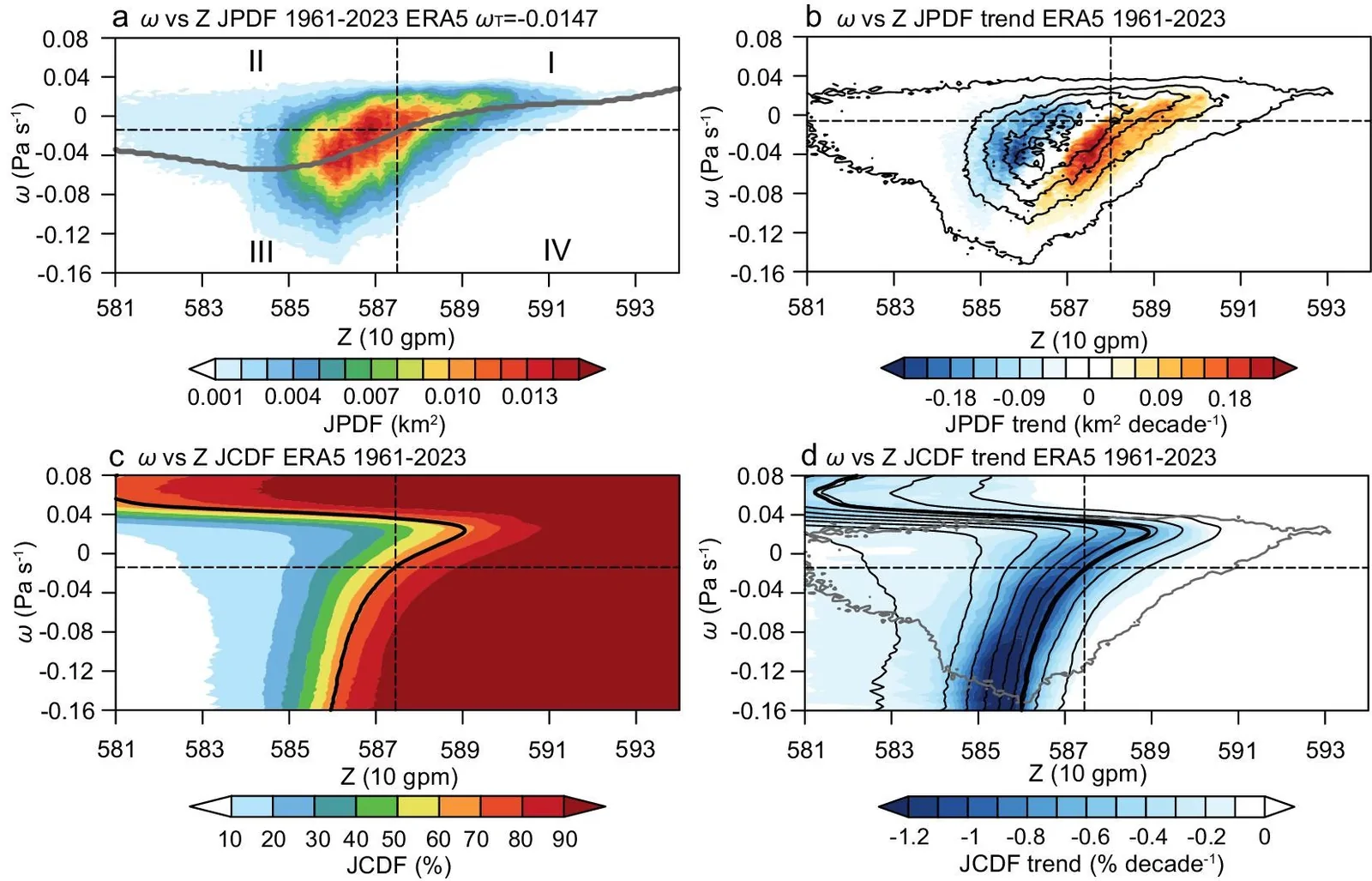
(a) Z-ω JPDF averaged in 1961–2023 based on ERA5 over Eastern Asia. The superimposed gray line is the mean ω value across all grid points at 500 hPa as a function of every 1 gpm Z bin. The value ω_T_ = −0.0147 Pa/s above the panel indicates the mean ω value across all grid points when Z = Z_T_ (vertical dashed line), which is superimposed as a horizontal dashed line. (b) Mean [same as (a), contour] and trend (shading, only trends significant at the 95% confidence level are plotted) of the Z-ω JPDF from 1961–2023 based on ERA5. (c) Z-ω JCDF averaged in 1961–2023 based on ERA5 over Eastern Asia. Thick black solid curves represent the JCDF_T_ (68.3%) contour lines of the JCDF, smoothed using a 9-point running mean filter [the 68.3% derives from the cumulative probability at the (Z_T_, ω_T_) point]. (d) Mean [same as (a), contour] and trend (shading) of Z-ω JCDF from 1961 to 2023 (per decade, only trends significant at the 95% confidence level are plotted). The contour represents the Z-ω JPDF at a value of 0.001 km^2^.

Next, we further clarify the relationship between the WPSH and Z-ω based on their JPDF. As shown in Fig. 2b, the increasing Z values do not necessarily lead to the same increase in the area that fulfills conditions favoring downward motion, suggesting that Z can increase due to thermodynamic expansion, even without a corresponding enhancement of circulation intensity. Since the impacts of WPSH are mainly attributed to its vertical airflow [4,23], and the magnitude and coverage of the downward motion within the WPSH are generally proportional to the WPSH intensity [4,7,24]. If the WPSH-associated ω remains constant, the dynamical intensity of WPSH likewise remains unchanged. Therefore, even when Z itself undergoes an increase due to global warming, the Z value corresponding to the same level of downward motion can still be used to delineate the WPSH.

As such, one can use ω as a dynamical constraint to determine the Z threshold for defining the WPSH. As mentioned above, the overall shift of the JPDF toward higher Z is primarily due to warming-induced thickening in geopotential height, rather than as an intensification or expansion of the WPSH. In this sense, one can identify time-varying Z values associated with comparable downward motion by examining the Z-ω JPDF, which provides a mapping relationship between Z and ω. This approach effectively removes warming-induced thickening trend in Z and allows the WPSH to be identified consistently under different climate backgrounds.

Since the conventional 5875 gpm contour is able to distinguish WPSH-related vertical motion in the present climate, here we use the probable ω value conditional on Z_T_ = 5875 gpm as a benchmark to evaluate how the WPSH boundary shifts from year to year. Specifically, in ERA5, grid points with Z_T_ = 5875 gpm correspond to a mean vertical velocity of ω_T_ = −0.0147 Pa/s (Fig. 2a). This ω_T_ is therefore used as the vertical-motion criterion associated with the WPSH.

To check whether this benchmark ω_T_ remains valid under a shifting climatic state caused by warming, we have conducted sensitivity analyses using ω_T_ derived from different previous periods. Although the Z-ω relationship (thick lines in Fig. 3a) shifts upward due to the long-term increase in Z, the slope of Z-ω (thin lines in Fig. 3a) remains similar across different climate states. This indicates ω_T_ represents a physically meaningful dynamical threshold rather than a purely statistical value. It corresponds to a fundamental dynamical structure (Fig. S3a; i.e. the sufficient descending motion required to produce the typical weather impacts) of the WPSH regardless of the climate states, characterized by convective suppression, clear sky conditions over East Asia. Under global warming, any overall increase or decrease in the ω_T_ area is a direct manifestation of changes in the extent of the WPSH itself. For more details about the motivation for choosing (Z_T_, ω_T_), the interpretation of JPDF on WPSH, and the sensitivity tests of JPDF in different periods, pressure levels and datasets, are provided in ‘JPDF’ of ‘Methods.’

**Figure 3.**
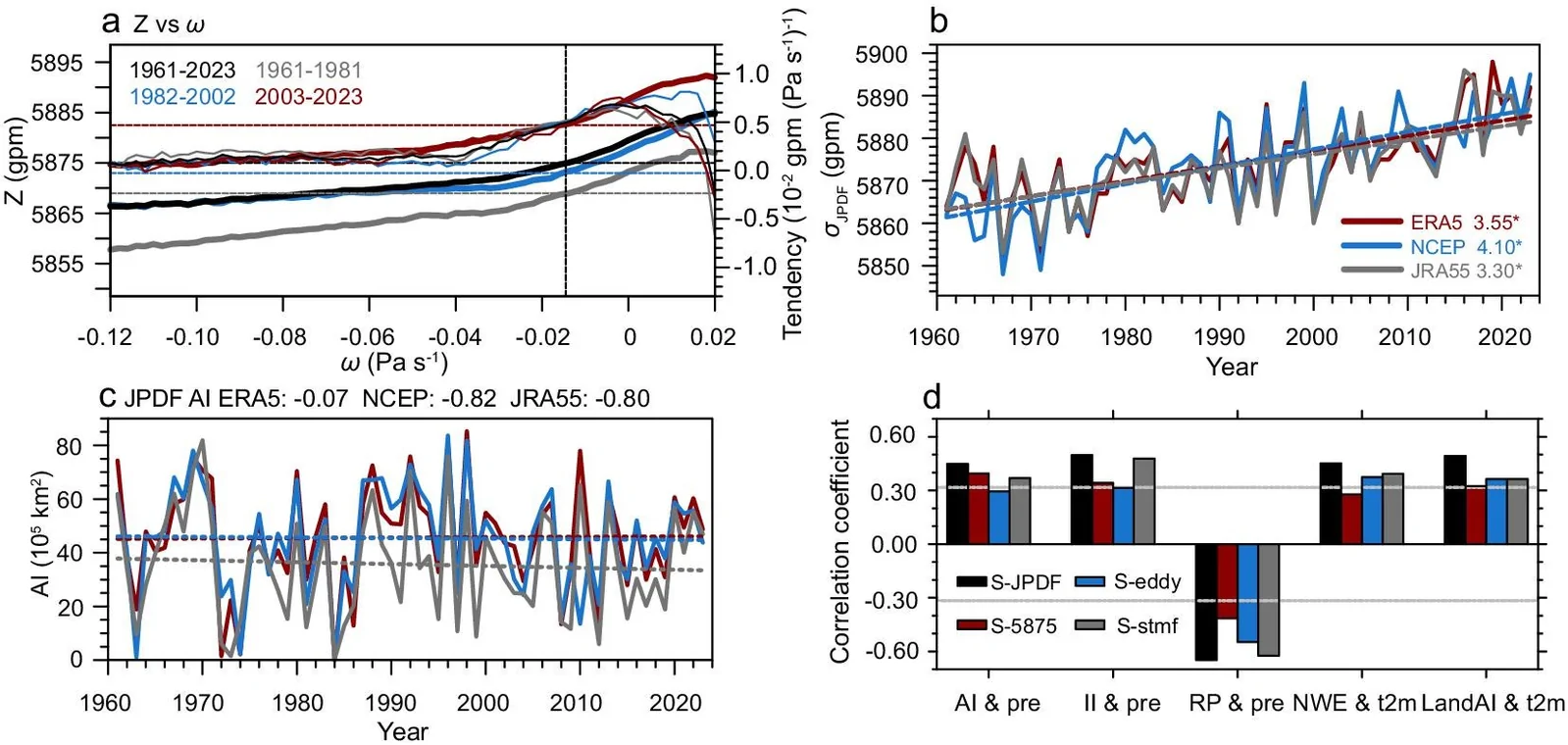
(a) Curve (thick solid lines) of Z (unit: gpm) against ω (unit: Pa/s) averaged over 1961–1981 (gray), 1982–2002 (blue), 2003–2023 (red), and 1961–2023 (black), based on ERA5 over Eastern Asia. The thin solid lines are the slopes [first derivative, unit: 10^−2^ gpm/(Pa/s)] of the 9-point running mean filtered curves. The thin vertical black dashed lines denote ω_T_, the averaged ω when Z = Z_T_. The colored thin horizontal dashed lines show the mean Z value for different periods when ω = ω_T_. (b) The annual evolution and trend (unit: gpm/decade) of σ_JPDF_ across different datasets (ERA5 in red, NCEP1 in blue and JRA55 in gray). (c) Temporal evolution of the AI indices (units: 10^5^ km^2^) of WPSH defined by S-JPDF. The red, blue, and gray lines represent results from ERA5, NCEP1, and JRA55 datasets. The trend of each reanalysis is shown above the panel (per decade), and all trends are insignificant at the 99% confidence level. (d) Correlation coefficients between different indices and temperature/precipitation under different WPSH strategies based on ERA5, with the solid gray line indicating the 95% significance threshold for the correlation coefficient.

To estimate the boundary of WPSH (σ_JPDF_), we technically used joint cumulative distribution frequency (JCDF; see the Method section) to project a Z value that favors downward airflow year to year. The Z-ω JCDF can be derived directly from the Z-ω JPDF and indicates the area where a certain Z occurs below a ω value (Fig. 2c). The JCDF shows an overall rightward shift in the JCDF pattern, indicating a Z-value increased from 1961 to 2023 (Fig. 2d). In ERA5, the climatic JCDF at (Z_T_, ω_T_) is 68.3%. This threshold implies that, for grid points with ω_T_, the upper 31.7% of the Z distribution exceeds Z_T_, corresponding to WPSH-like characteristics. Based on the JCDF_T_, a non-fixed Z value at ω_T_ can be derived in each year, and we refer to it as the threshold (σ_JPDF_) for defining the boundary of the WPSH. As the air column warms, σ_JPDF_ in ERA5 increases obviously with increasing temperature, with the growth rate of σ_JPDF_ being 3.55 gpm/decade (Fig. 3b). This suggests that σ_JPDF_, defined as the dynamically constrained Z threshold under ω_T_, incorporates the warming-induced increase in Z and therefore provides a physically consistent metric for describing the WPSH boundary in a warming climate. The WPSH defined by the above method will be referred to as S-JPDF hereafter.

### Advantages of S-JPDF for WPSH

Since the WPSH is a key indicator influencing East Asian climate, an effective WPSH definition should be able to capture the associated climate anomalies. Next, we compare the correlations between different WPSH definitions and temperature/precipitation, to examine the practicability of the WPSH as defined by S-JPDF in diagnosing weather and climate anomalies, as well as its advantages over previous definitions in practical application. Averaged over 1961–2023, the spatial WPSH coverage in S-JPDF (red solid line in Fig. 1d) is similar to that in S-5875, showing a good consistency with the location and morphology of the rainband. This suggests its potential in weather and climate diagnosis.

The composite results of climate and weather anomalies (Fig. 4) demonstrate that the WPSH characterized by S-JPDF can well explain the occurrences of both extreme precipitation and heatwaves. In precipitation cases (Figs 4a–c, S5a–j, S6), it is found that the WPSH boundary in S-JPDF corresponds to the rainbands and upward airflow zone on its northwest side, which implies it may have good performance in the diagnosis of the locations of precipitation anomalies. A similar composite analysis is conducted on the temperature anomalies. It is found that the WPSH coverage in S-JPDF matches well with the downward airflow that favors high temperatures (Figs 4d–f, S5k–u, S7). The above results illustrate that the WPSH in S-JPDF can provide more physically robust diagnostic information for extremes regardless of whether it is on seasonal or synoptic scales. For more details about the spatial relationship between extremes and WPSH in S-JPDF and the comparison with previous metrics, readers can refer to the ‘Methods’ section.

**Figure 4.**
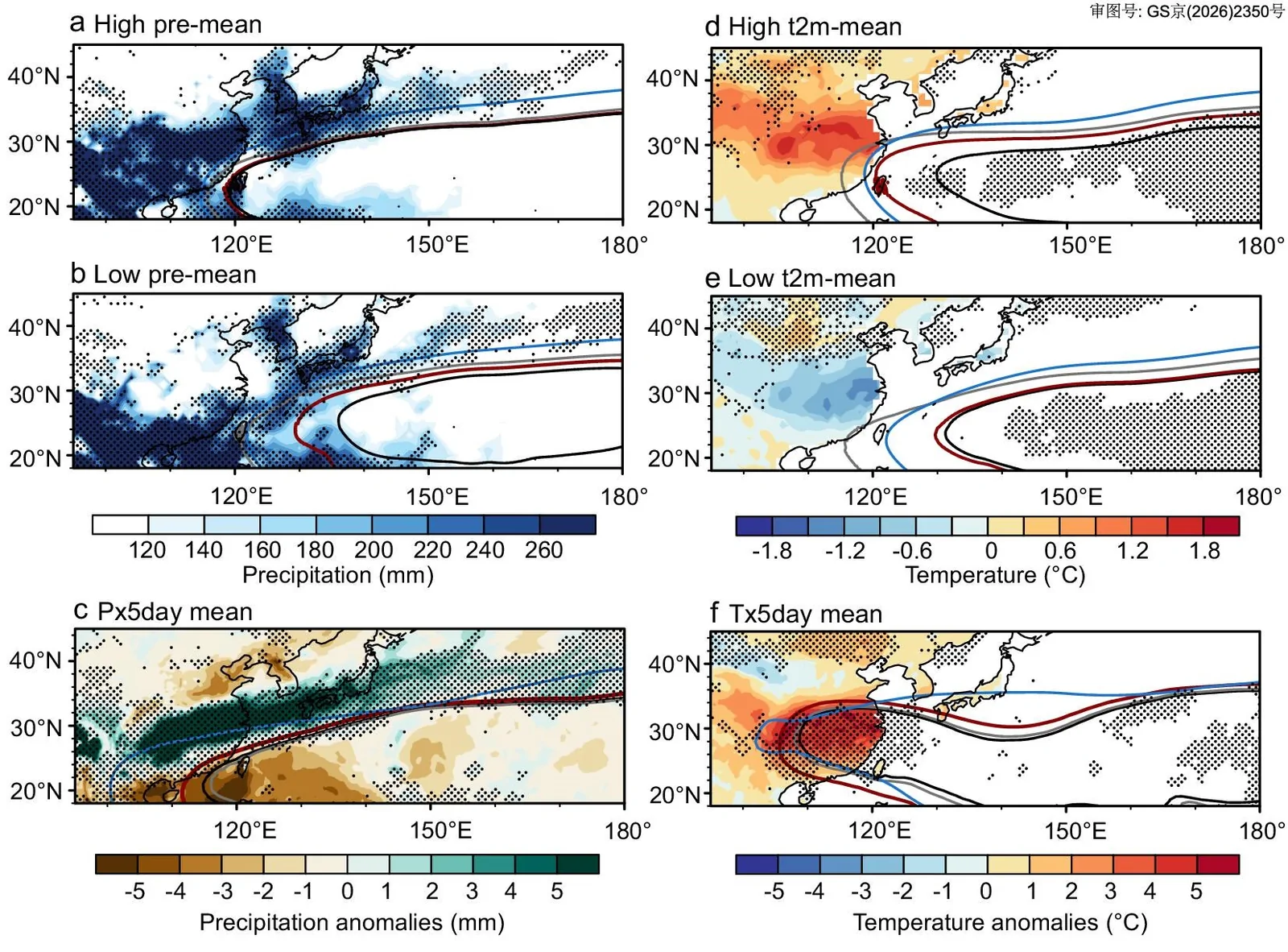
Distribution of precipitation against the WPSH boundary averaged in (a) 5 wettest summers, (b) 5 driest summers, and (c) the spatial distribution of Px5day (5-day running-mean precipitation) anomalies and WPSH boundaries during the top 16 extreme precipitation events across different WPSH definitions. Distribution of 2 m air temperature against the WPSH boundary averaged in (d) 5 hottest summers, (e) 5 coldest summers, and (f) the spatial distribution of Tx5day (5-day running-mean 2 m temperature) anomalies and the WPSH boundaries during the top 16 extreme heat events from 1961 to 2023 across different WPSH definitions. The five wettest, driest, hottest, and coldest summers were selected using area-averaged precipitation and temperature anomalies over the Sichuan-Yangtze Basin (26°N–33°N, 104°E–121°E). Extreme precipitation and heat events were identified using Px5day and Tx5day exceeding 1.96 standard deviations over the Sichuan-Yangtze Basin. Dotted shading in (a–c) and (d–f) shows the upward airflow (ω < −0.0147 Pa/s) and downward airflow (ω ≥ −0.0147 Pa/s), respectively. The black, red, blue, and gray solid lines represent the WPSH boundary in the ERA5 dataset in S-5875, S-JPDF, S-eddy, and S-stmf definitions, respectively.

To quantify the ability of the WPSH in S-JPDF in diagnosing temperature and precipitation in East Asia, correlation analysis is used between the long-term variations of the WPSH indices and temperature/precipitation. Following previous studies, the AI (Area index), II (Intensity index), and RP (Western Ridge Point index) are three widely used canonical indices for precipitation diagnosis [14,25,26] (Fig. 3d). In S-5875 and S-stmf, the three indices exhibit significant correlations with precipitation series in eastern China (at the 99% confidence level), while the highest correlation coefficient is found in S-JPDF. For temperature-related impacts, which are more sensitive to the landmass coverage and northwestward expansion of the WPSH [1,27], we developed two additional indices (details are provided in ‘WPSH Indices’ of ‘Methods’): the Northwest Extension Index (NWE) and Land Area Index (LandAI). Significant correlations (at the 99% confidence level, Fig. 3d) are observed between the two indices and the temperature series in S-JPDF, S-eddy, and S-stmf. Among these three definitions, S-JPDF exhibits the highest correlation coefficient. These results imply that the WPSH measured by S-JPDF is well able to characterize the weather and climate anomalies.

As described in section ‘Disagreements in WPSH’ in ‘Methods,’ the inconsistency in the long-term trends of the WPSH under the previous definitions and different datasets has led to significant uncertainties in the WPSH and its impacts. Next, we investigate the long-term trends in WPSH based on the S-JPDF. As shown in Fig. 3c and Fig. S8a, the trends of the AI and II across different datasets are consistent, characterized by a weakening from 1961 to 1980, followed by a strengthening after 1980. This is associated with the inter-decadal shift of the WPSH in the 1980s [16,22]. The RP has exhibited a westward retreat and then an eastward extension, which matches the trend in AI and II. The trends of the AI, II, and RP are insignificant over the entire period, indicating that the WPSH has not been significantly affected by warming over the past decades. More importantly, the trends of the WPSH show a higher agreement across different datasets than those from previous definitions, which implies the trends derived from S-JPDF are more credible. Previous studies have found that there are slight differences in climatological means of Z in different reanalysis datasets, which are due to the sources and time of satellite observation data used, as well as systematic model biases [28,29]. Both the S-eddy and S-stmf require the use of climatological means to correct the impact of warming. In contrast, the S-JPDF is directly based on the geopotential height that is synergistic with changes in ω and thus is not affected by biases from the climate states. This may explain the high agreement in the trend obtained from S-JPDF.

The above results illustrate that the WPSH defined by the S-JPDF has a good spatial match with precipitation and temperature anomalies in both weather and climate scales, and also exhibits high agreement in long-term trends. The WPSH defined by S-JPDF exhibits similar spatial coverages with S-5875 in climate states, while notable discrepancies occur between them in terms of the annual evolution. This suggests substantial differences between the two definitions. Additionally, the definitions- and datasets-dependence of the spatial coupling with weather anomalies and the long-term trends in S-eddy and S-stmf introduce uncertainties in the analysis of the WPSH. These findings suggest the necessity of taking the response of thermodynamic-dynamic synergy into account when studying the weather diagnosis and the long-term change of the WPSH.

### The WPSH trends in the CMIP6 historical simulations

Although σ_JPDF_ changes over time in the past six decades, its value generally varied from 5850 to 5890 gpm (Fig. 3b), which agrees with the range of traditional fixed contours of WPSH boundary (5860–5880 gpm contour) [27, 30, 31]. Thus, the impact on historical WPSH analyses may not be crucial. Obviously, σ_JPDF_ will further increase, reaching 5900 gpm or higher, in the 21st century based on climate model projections. Using S-JPDF to study the future changes of WPSH makes more physical sense. Thus, in the following, we further examine the future changes of the WPSH under different Shared Socioeconomic Pathway scenarios (SSP1-2.6, SSP2-4.5, SSP3-7.0 and SSP5-8.5), based on the climate projections by 26 CMIP6 models (see Method section). Before that, however, we first evaluate the model performance on the historical changes in σ_JPDF_ and WPSH.

In S-JPDF, we adopt the probability of the Z_T_ to define the WPSH in reanalysis datasets. However, considering the model systematic bias of Z, directly using the probability of the Z_T_ in CMIP6 may not be a good choice. Figure S9a gives the uncertainty range for Z and ω. The climatological Z values in East Asia from 26 CMIP6 models range from 5794 to 5893 gpm (the blue bar), which deviates from the observed 5852 gpm (the grey shaded). Thus, we employed a linear correction method (see ‘Linear scaling correction’ in the ‘Methods’ section) to correct the climatological bias in Z. Without such a correction, most models cannot identify the WPSH using the definition adopted in this study. After correction, the Z values in CMIP6 models (the red bar) are generally comparable to observations. Only the corrected Z in CMIP6 will be used hereafter. No correction was applied to ω, since its values (the green bar) are consistent with observations.

We first evaluate the ability of CMIP6 models to simulate the relationship between Z and ω in the historical period. The Z-ω JPDF pattern and averaged ω across different Z-value bins in the Multi-Model Ensemble (MME) show a similar distribution to that in observations (Fig. S9b). The distribution of frequency in the first quadrant (Fig. S9c), separated by (Z_T_, ω_T_), agrees with observations (Fig. S3a), which indicates the ability of MME to reproduce the characteristic of WPSH. MME also captured the rightward shift trend in the Z-ω JPDF and JCDF (Fig. S9b, d). Moreover, the σ_JPDF_ value derived from the JCDF exhibits a significant increasing trend from 1961–2023, with a growth rate of 4.90 gpm/decade (Fig. 5a). This value is slightly higher than the observed, which is attributed to CMIP6 models’ higher sensitivity to atmospheric warming in mid-latitude [32].

**Figure 5.**
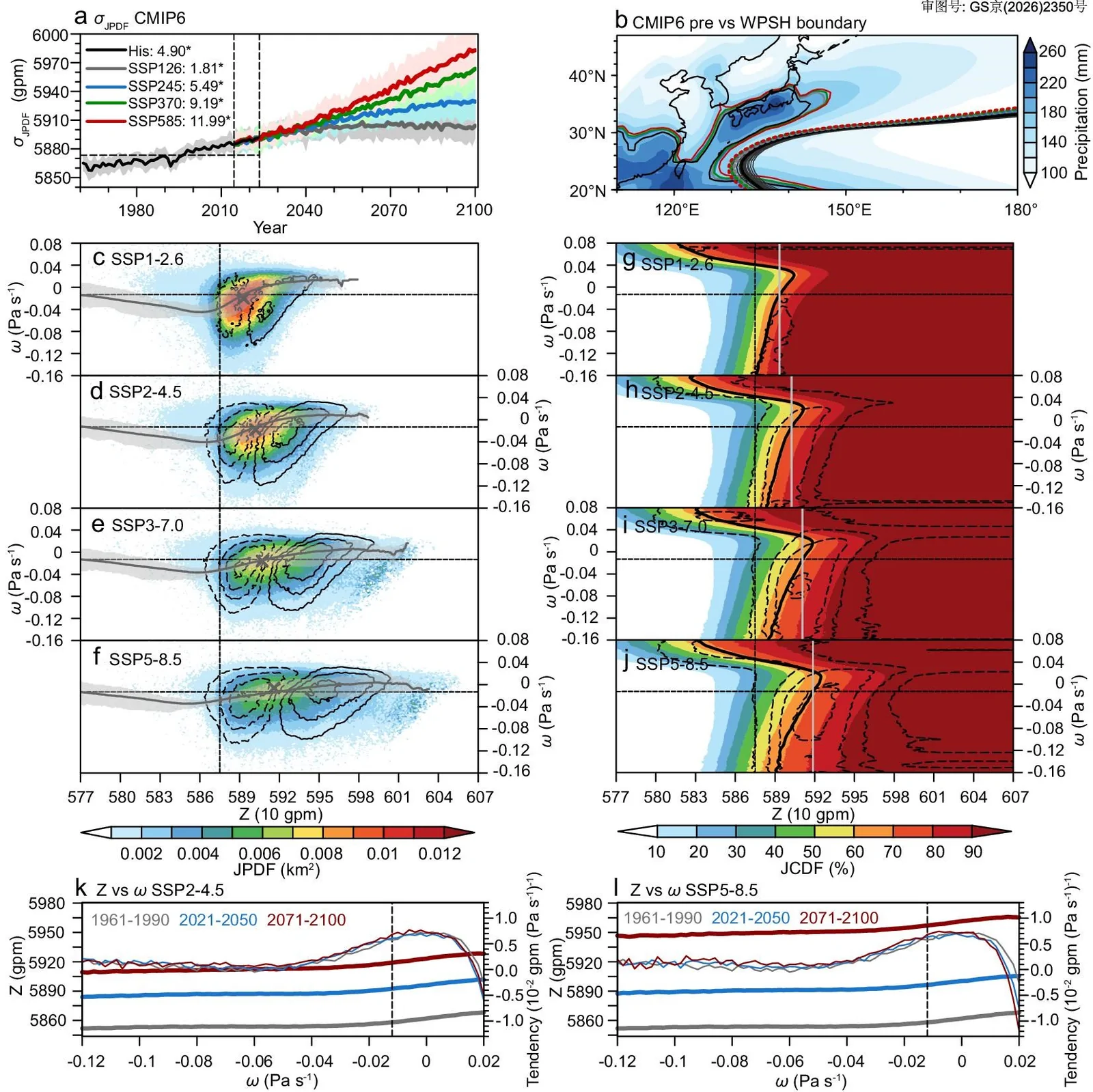
(a) The annual evolution and trend (units: gpm/decade) of σ_JPDF_ in MME, with shading showing the uncertainty range of individual models. (b) The boundary of the WPSH (contours) against the distribution of monthly mean precipitation averaged over all SSPs (shading) in CMIP6. Black and gray solid lines show the results of the MME and individual models in 1961–2023, and the gray, blue, green, and red dashed lines represent the results for SSP1-2.6, SSP2-4.5, SSP3-7.0, and SSP5-8.5 scenarios from 2024 to 2100. The thin solid lines of corresponding colors outline the summer rainfall amount of 180 mm/month contours for the historical period 1961–2023 and the SSPs period 2024–2100, respectively. (c–f) Z-ω JPDF in Eastern Asia averaged over the period 2024–2100 from the MME of CMIP6. From top to bottom, the panels represent the Z-ω JPDF under SSP1-2.6, SSP2-4.5, SSP3-7.0, and SSP5-8.5 scenarios, respectively. The superimposed gray line and shading denote the mean ω value across all grid points at 500 hPa as a function of every 1 gpm Z bin, derived from the CMIP6 MME and individual models. The black lines depict the trends, with positive trends for solid lines and negative trends for dashed lines. The × marks the location of the maximum value within the JPDF. (g–j) Same as (c–f) but for the Z-ω JCDF instead. (k, l) Same as Fig. 3a but for curves of Z against ω averaged for 1961–1990 (gray), 2021–2050 (blue), and 2070–2100 (red) under SSP2-4.5 and SSP5-8.5 scenarios.

In the MME, the westernmost longitude of the WPSH is 131.3°E, with individual models varying within a range of 128.4°E to 133.8°E (Fig. 5b). This range is consistent with the observed 131.6°E. Additionally, the northwestern side of the WPSH matches well with the summer rainband. From 1961 to 2023, there is no significant trend of AI, II, and RP in MME, which agrees with the observations. Most models (24 out of 26) give no statistically significant trends of the WPSH, except for CMCC-CM2-SR5 and MPI-ESM1-2-LR. The aforementioned analysis indicates that the WPSH features defined by the S-JPDF are well reproduced by CMIP6 models, enhancing the confidence of future projections.

### CMIP6 projection of WPSH’s future changes

As the troposphere continues to warm, the applicability of the S-JPDF method depends on whether the statistical linkage between Z and ω remains stable. Therefore, we check the Z-ω relationship in future projections. Although the absolute value of Z increases with warming, the slope of ω-Z relationship remains consistent across different scenarios and periods (Figs 5h, i and S9e, f). This indicates that the more scattered future JPDF is caused by the systematic increase in Z, rather than by a fundamental change in the mapping between ω and Z. This result is consistent with the historical analysis (Fig. 3a) and supports the use of the present-climate reference JCDF in the S-JPDF method.

We next assess the projected pattern of the Z-ω JPDF and its influence on σ_JPDF_ under different emission scenarios. Figure 5c–f exhibits Z-ω JPDF distribution under the different SSPs in MME for 2024–2100. It projects a long-term rightward shift of the Z-ω JPDF, with a larger shift and more visible spread under higher emission scenarios. Under the SSP1-2.6 scenario, the maximum JPDF (marked as ×) lies below ω_T_. The maximum JPDF exceeds the ω_T_ in higher scenarios, indicating more frequent occurrence of downward airflow. The distribution of the Z-ω JCDF averaged over 2024–2100 also exhibits a notable rightward shift compared to the historical period [compared to (Z_T_, ω_T_)]. The higher the emission scenarios, the more evident the rightward shift. Specifically, under SSP1-2.6, the Z-value averaged in 2024–2100 is 5900.1 gpm when ω = ω_T_ and JCDF = JCDF_T_, while this value increases to 5919.5 gpm under SSP5-8.5. Consequently, the σ_JPDF_ shows a significant increase in the future, with more pronounced growth under stronger emissions (Fig. 5a).

Compared to the historical period, the future averaged WPSH boundary exhibits a westward expansion, as well as an increased coverage of the WPSH (Fig. 5b), which indicates an enhanced WPSH. Under SSP1-2.6, the trends in AI and II are not significant. However, under higher scenarios, both indices exhibit an evident increase, with the higher growth rate under stronger scenarios (Figs 6a–c and S10a, b). Meanwhile, significant westward extensions of RP are found across SSPs, with the most pronounced trend under SSP2-4.5. Considering that AI, II, and RP serve as diagnostic indicators for the intensity and location of rainband, the aforementioned changes of WPSH may suggest an intensification and a westward expansion of the summer precipitation in the future. Temperature-related land exposure is then assessed using the NWE and LandAI indices. Both indices show increasing trends under SSPs, with a more evident growth rate under higher emissions. These results suggest that the WPSH will shift northwestward, causing larger land areas in East Asia to be affected by WPSH-related subsidence and associated heat conditions.

**Figure 6.**
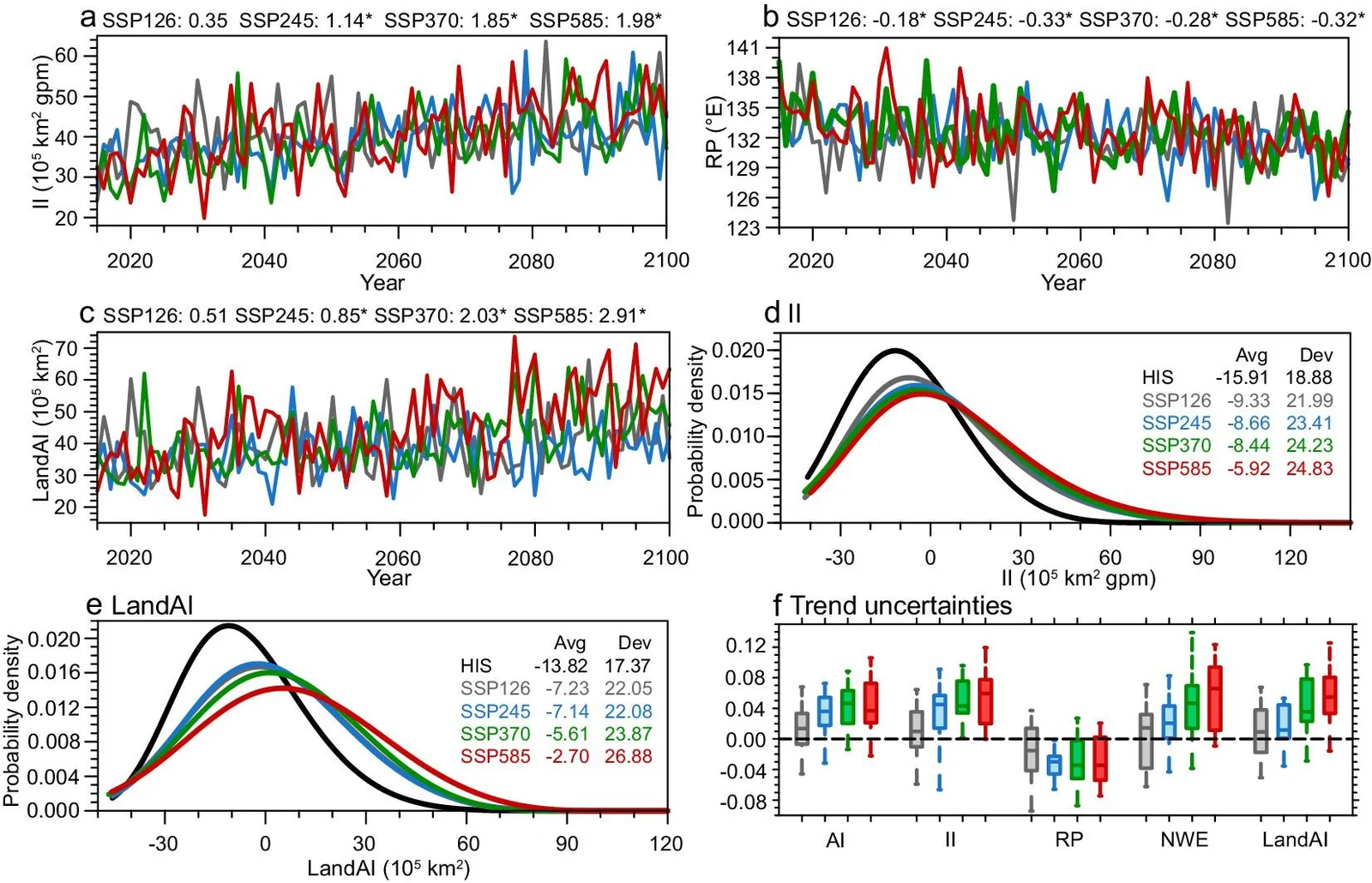
Temporal evolution of simulated (a) II (units: 10^5^ km^2^ gpm), (b) RP (units: °E), and (c) LandAI (units: 10^5^ km^2^) indices of WPSH in MME over 2015–2100. The trend of each scenario is shown above the panel (per decade), and ‘*’ denotes significance at the 99% confidence level. The PDF of (d) II and (e) LandAI anomalies for the historical (1965–2014) and SSPs (2051–2100) run. The digits in the fig. (d, e) represent the average (Avg), standard deviation (Dev) of the corresponding period. The gray, blue, green and red lines represent the results of SSP1-2.6, SSP2-4.5, SSP3-7.0, and SSP5-8.5. (f) The box-and-whisker panel of the projected trend range of standardized WPSH indices. The box is spanned by the 25th, 50th and 75th quantiles (lower, median and upper border of the box) and 5th and 95th percentiles (lower and upper edges of vertical dashed whiskers) across the models.

The overall shift in the PDF of WPSH indices also exhibits an intensification along with an expansion of WPSH, as the mean value is projected to increase (decrease in RP). More importantly, the increased standard deviation and the rightward skew of high values in the long right tail (leftward skew in the left tail of RP) suggest a higher occurrence frequency of extremely strong WPSH events in the future (Figs 6d–e and S10c–e). To give the uncertainty of projection, Fig. 6f shows the range of trends for all 26 models in different WPSH indices and scenarios over 2024–2100. Under SSP2-4.5, SSP3-7.0, SSP5-8.5, more than three-quarters of the models agree with the MME trend for all five WPSH indices, and the higher the emissions, the more pronounced the trend, indicating that the MME’s projections are robust. Under SSP1-2.6, half of the models exhibit an increasing trend while the other half show a decrease, which implies uncertainty in the changes of the WPSH under this scenario.

The above WPSH analysis was conducted on the seasonal scale. However, on the synoptic scale, the WPSH exhibits greater spatiotemporal variability [5,27]. It can extend inland to the Tibetan Plateau and even connect with the Iranian High, resulting in a more complex morphology of the WPSH western side due to the influence of the inland terrain. Therefore, it is worthwhile exploring the daily change of the WPSH. Accordingly, we applied the S-JPDF to the daily geopotential height data to measure the WPSH (only AI, II, and LandAI were focused; the reason for excluding RP and NWE is detailed in ‘WPSH Indices’ of ‘Methods’). This daily-scale analysis is conducted using only 15 out of the 26 models under SSP2-4.5 and SSP5-8.5 (marked with an asterisk in Table S2), as daily geopotential height data were available for only these 15 models. For each index, abnormally strong WPSH cases were defined as those satisfying two criteria: (1) exceeding 1.96 times the standard deviation (corresponding to the 95% confidence level), and (2) persisting for more than three consecutive days.

Figure 7a–c presents the MME of the spatial composite for the top 10 and top 20 cases of abnormally strong WPSH under different scenarios. The WPSH morphology derived from the AI is distinct from that from the II. Specifically, the WPSH associated with AI extends more inland, with the westernmost ridge point of the composite of the top 10 cases reaching 105.8°E in the historical period. In contrast, the WPSH morphology based on the II exhibits a smoother shape, with the western ridge point located around 110°E. The LandAI index yields a distinct morphology compared to the above two indices, with the ridge point of the top 10 cases extending westward to near 90°E. Under SSP2-4.5 and SSP5-8.5, the composites of the top 10 and top 20 WPSH cases of the three indices all show a significant westward extension. This extension is more pronounced under the SSP5-8.5 than under the SSP2-4.5, which is consistent with the conclusions from seasonal data. A critical finding is that the composite based on LandAI suggests an increased likelihood of the WPSH connecting with the Iranian High in the future. This phenomenon may trigger a cascade of feedback processes and lead to more extreme weather events that would surpass historical records.

**Figure 7.**
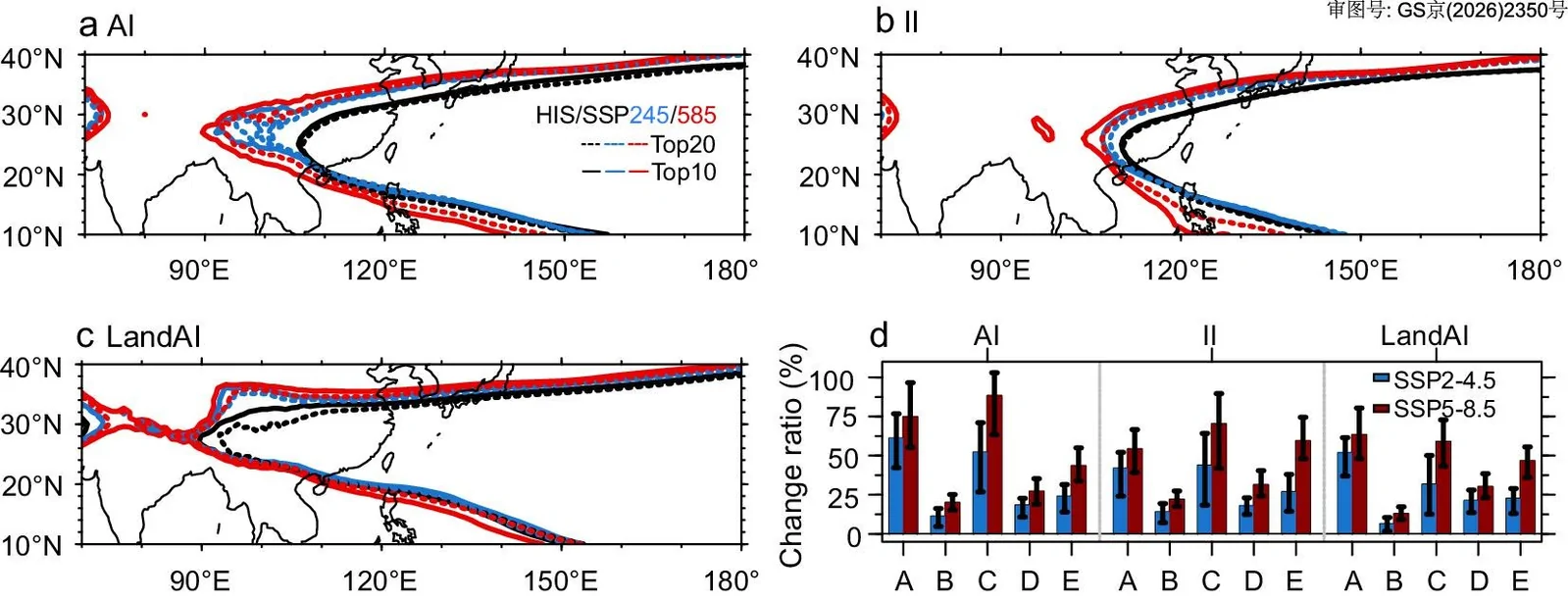
MME of the spatial distribution composite of abnormally strong WPSH events. Abnormally strong WPSH events were selected when their values exceeded the 95th percentile of the climatological daily series of (a) AI, (b) II, and (c) LandAI. The black, blue, and red lines represent the results of the historical (1961–2014), SSP2-4.5 (2015–2100), and SSP5-8.5 (2015–2100), respectively. Solid and dashed lines represent the composites of the top 10 and top 20 abnormally strong WPSH events, respectively. (d) The growth rates of the annual frequency (A), average duration (B), maximum duration (C), average intensity (D), and maximum intensity (E) of all abnormal WPSH events under SSPs are calculated relative to the historical run. The whisker bars represent the uncertainty range of individual models.

We further quantified the change ratios for all abnormal WPSH cases under SSPs (Fig. 7d). The strong WPSH cases reveal consistent increasing trends across their occurrence frequency, average duration, maximum duration, average intensity, and maximum intensity, based on the AI, II, and LandAI. Notably, the growth is more pronounced under higher emission scenarios, and almost all individual models agree on this growth. The most significant growth is observed in the occurrence frequency and maximum duration of strong WPSH (columns A and C), with increases exceeding 30% for all indices under both emission scenarios. This implies that in the future, strong WPSH cases will not only become more intense, but also exhibit markedly longer durations and higher frequency.

## DISCUSSION AND CONCLUSION

Early studies developed either thermodynamic or dynamic definitions to avoid inadequacy in traditional measurement of the WPSH. However, the changes of the WPSH described by these definitions diverge both in the past and the future. This is attributed to the definition metrics of WPSH treating its thermodynamic or dynamic aspects separately, leading to disagreements in their characterization of the WPSH response to climate change. Here, by developing a comprehensive S-JPDF definition that considers the WPSH thermodynamic-dynamic synergy, we find that it can provide more physically coherent information for diagnosing weather anomalies related to the WPSH. And the trends of WPSH indices in S-JPDF are consistent across datasets, which indicates a high certainty in their changes.

In the future climate, regardless of the SSP scenario, our results show robust increases in area and intensity of WPSH, and the boundary of WPSH will extend northwestward, accompanied by an increase in landmass coverage, which are consistent across datasets and supported by a strong inter-model consensus. Particularly, climate models project an increased likelihood of record-breaking WPSH events on both seasonal and synoptic scales, with stronger intensity, longer durations, and higher occurrences under higher-emission scenarios. Given that the WPSH is a key indicator for the intensity and location of rainband, these results may suggest an intensification and a northwestward expansion of WPSH-related summer flood and heatwave risks in East Asia in the future [33–36].

It should be noted that earlier studies revealed the spurious strengthening of the WPSH defined by S-5875, therefore several optimized definition metrics for WPSH have been developed [8,13,14,17,25]. This study compares the differences among S-JPDF, S-eddy, and S-stmf, validating the advantages of S-JPDF in linking the WPSH to weather anomalies, as well as in capturing long-term trends. Nevertheless, S-eddy and S-stmf also possess their respective strengths. Specifically, S-eddy interprets the WPSH from a thermodynamic perspective, regarding it as a deep, warm, and dry high-pressure system. This method provides a robust framework for studying the WPSH-rainband linkages. However, it exhibits limitations in depicting the WPSH’s evolution due to its reliance on the changing climate states [7,13,37]. In contrast, S-stmf defines the WPSH as a dynamic anticyclonic circulation, which is independent of atmospheric column thickening [38]. This definition does not account for the influence of latent heat release on the WPSH’s structure, thereby limiting its ability to establish correspondence with rainband positions [17,21,31]. Given that the thermodynamic and dynamic processes of the WPSH may respond differently to warming, it is essential to take both processes into account when studying WPSH.

Much of the recent literature continues to rely on the traditional S-5875 definition. For example, the coverage of the 5880 gpm contour was employed to diagnose heatwave [1,39–42]; the interannual variation of the 5870 gpm contour was used to study extreme precipitation [27,43–45]. The widespread adoption of S-5875 in earlier research is attributed to its ability to maintain physical connections with extreme weather under the current climate. This is the most significant trait of WPSH in operational practice, which is preserved in S-JPDF. Therefore, S-JPDF is a more robust and practical approach for assessing both the weather impact of WPSH and its response to climate change.

One may be concerned that the WPSH trend characterized by the S-JPDF appears to behave more similarly to that represented by the dynamic-based metric. However, this does not imply that dynamic factors necessarily dominate the behavior of the WPSH. The long-term changes of the WPSH are regulated by multiple thermodynamic and dynamic processes. Under global warming, changes in the tropical meridional SST gradient and Hadley circulation, enhanced Tibetan Plateau heating, increased land-sea thermal contrast and northward displacement of the westerly jet, enhanced latent-heating feedbacks associated with increased atmospheric moisture, and a stronger atmospheric response to La Niña-like SST anomalies [19,46] may together modulate the intensity, zonal and meridional extensions of the WPSH. The relative roles of dynamic and thermodynamic factors in shaping the WPSH require more detailed diagnostic analyses. Moreover, the present 500-hPa WPSH study does not address its three-dimensional structural changes, while preliminary results suggest that the S-JPDF is also applicable at 700 and 850 hPa (Fig. S2e, f). Another caveat concerns the assumed stability of the Z-ω relationship used in the S-JPDF. The historical reanalyses and CMIP6 simulations here consistently show an approximately stable slope of the Z-ω relationship, suggesting that the present-climate JCDF reference remains applicable for diagnosing future WPSH changes. However, this should be regarded as an empirical robustness result rather than a physically guaranteed invariance. Under more extreme warming climate states with substantially different convective organization, cloud-radiative feedbacks or land-sea thermal contrast, the coupling between geopotential height and vertical motion may change [47,48]. Such changes could affect the σ_JPDF_ threshold and hence the inferred WPSH boundary. Future studies using thermodynamic-dynamic process-based diagnostics are needed to further test the applicability of S-JPDF under a broader range of climate regimes. These issues will be further investigated in our future work.

The WPSH response to greenhouse warming is an important topic for projecting future changes in the global climate system. The responses should be regarded as one circulation-driven contributor to the projected increase in regional hydroclimate extremes, particularly the increased frequency, duration, and coverage of heatwaves, extreme precipitation, and their compound events in China [34–36]. Given that the most direct effect of the WPSH on the East Asian climate system is its role in modulating atmospheric vertical motions and water vapor transport, along with associated impact and feedback [49,50], it is essential to incorporate S-JPDF in studying the long-term change of the WPSH and its effects on weather and extreme events in a warming climate.

## METHODS

Detailed descriptions of methods are available in the online Supplementary Data.

## Supplementary Material

nwag497_Supplemental_File

## Data Availability

The authors acknowledge the FAIR data policy. Data relevant to the paper can be downloaded from the websites listed below. The ERA5 reanalysis products are available at: https://cds.climate.copernicus.eu/datasets. NCEP/NCAR reanalysis products are available at: https://www.esrl.noaa.gov/psd/data/gridded/data.ncep.reanalysis.derived.html; JRA-55 reanalysis products are available at: https://jra.kishou.go.jp/JRA-55/index_en.html; The outputs of CMIP6 models are available at: https://esgf-node.llnl.gov/projects/cmip6/. The WPSH boundary dataset based on the S-JPDF method described in this paper can be found in the repository at https://github.com/YenhsinC/WPSH_boundary_by_JPDF. All codes for the analysis are available upon request to the corresponding authors in this paper.
