# Supplementary material for: Anthropogenic climate change is causing stronger and more extreme western Pacific subtropical high
Source: Natl Sci Rev. 2026 Aug 17;13(18):nwag497. doi: 10.1093/nsr/nwag497 (PMC13625014; doi:10.1093/nsr/nwag497)
Supplement: Supplementary file 1 [file nwag497_supplemental_file.docx]

Supplementary Data for

**Anthropogenic climate change is causing stronger and more extreme Western Pacific Subtropical High**

Yanxin Zheng, Jeremy Cheuk-Hin Leung, Shuanglin Li^*^, Banglin Zhang^*^

*Corresponding author Email: [shuanglin.li@cug.edu.cn](mailto:shuanglin.li@cug.edu.cn) and [zhangbanglin24@nudt.edu.cn](mailto:zhangbanglin24@nudt.edu.cn) .

**This PDF file includes:**

Methods

Figures S1 to S11

Tables S1 to S3

References for Supplementary Data

**Methods**

**Data**

To assess the sensitivity of different WPSH definitions to datasets. Three monthly and daily reanalysis datasets from 1961-2023 were used, including the ERA5 with a 0.25° × 0.25°, JRA55 with a 1.25° × 1.25° grid and NCEP1 with a 2.5° × 2.5° grid horizontal resolution. Previous studies regarding the long-term changes of WPSH are generally based on the three datasets. Besides, the datasets vary in their resolution, which allows us to understand the WPSH definition at different resolutions. Four variables, geopotential height (Z), vertical wind (ω), zonal wind (u), and meridional wind (v), are used to define the WPSH, and precipitation (pre) and 2m temperature (t2m) are used to diagnose the impacts of the WPSH.

Apart from observed data, the monthly outputs of 26 Coupled Model Intercomparison Project phase 6 (CMIP6) models were used in the analyses of the WPSH change in past and future climate conditions (Table S2). The choices of models depend on their availability of all four SSPs (1-2.6, 2-4.5, 3-7.0, and 5-8.5) scenarios and variables output at the time (from 1961-2100) of analysis. For the daily outputs, 15 of the 26 CMIP6 models (marked with an asterisk in Table S2) were used under SSP2-4.5 and SSP5-8.5 scenarios, as daily data of geopotential height was available for only these 15 models.

**WPSH Indices**

Quantifying WPSH requires giving its boundaries. To reduce ambiguity among the different metrics, we first introduce four the WPSH definition metrics: the conventional fixed geopotential-height metric S-5875, the thermodynamic-based S-eddy, the dynamic-based S-stmf, and the hybrid thermodynamic-dynamic S-JPDF (Table S3).

The conventional fixed WPSH boundary is commonly identified using the 5880 gpm contour of 500-hPa geopotential height. While for S-5875 in this study, we use the 5875 gpm contour as the threshold to ensure a closed WPSH boundary in each year ^1,2^. To avoid the biases of the WPSH in S-5875 caused by the overall rising of the air column due to warming, alternative thermodynamic- and dynamic-based definitions have been developed^3,4^. Previous studies have shown that thermodynamic-based definitions generally indicate a weakening of the WPSH, whereas dynamic-based definitions tend to indicate an enhancement. Thus, we employed the S-eddy as a representative thermodynamic-based metric, and the S-stmf as a representative dynamic-based metric. For both S-eddy and S-stmf, the 0 contour is used as the boundary threshold, while for S-JPDF, σ_JPDF_ is used.

Based on the threshold σ of WPSH boundaries of each metric within the East Asian (10°N-40°N, 90°E-180°E), namely σ_5875_, σ_eddy_, σ_stmf_, σ_JPDF_, we define the following WPSH indices ^5,6^:

(1) Area index (AI, units: 10^5^km^2^): the sum of the area of all grid points greater than or equal to the σ;

(2) Intensity index (II, units:10^5^km^2^·gpm): the sum of the product of the area of all grid points greater than or equal to the σ and the difference of the value relative to the σ;

(3) Western Ridge Point index (RP, units: °E): the westernmost longitude of σ contour;

The above canonical indices are closely related to precipitation, which are effective indicators of the occurrence of anomalous precipitation in East Asia, thus being widely used. However, there is no significant correlation between the three indices and the temperature anomalies, even though studies have demonstrated a close connection between the WPSH and the occurrence of heatwave events^7,8^. To address the issue, we developed two indices to study the ability of WPSH in examining temperature anomalies:

(4) Northwest Extension index (NWE, units: -): When the RP is anomalously westward, heavy precipitation or high temperature events occur in eastern China. However, at this time, if the westward extending ridge line (RL) of the WPSH is anomalously northward, it has a higher probability of a heatwave^9,10^. Therefore, we combined the PR and RL indices to characterize the northwestward expansion of WPSH. NWE is defined as:

NWE = (-1*std(RP))+ std(RL)

A larger NWE indicates a stronger northwestward expansion of the WPSH, and vice versa. The RL index is defined as the average value within two latitude degrees around the grid point where the value is equal to σ, the zonal wind speed is zero (u=0), and the wind field nearby exhibits anticyclonic rotation ("∂u/∂y=0") within the region^19^.

(5) Land Area index (LandAI, units: 10^5^km^2^): The land area covered by the WPSH tends to experience an anomalous high temperature, so LandAI is defined as the sum of the area of all grid points greater than or equal to the σ over the land area (20°N to 40°N, 90°E to 180°E).

It should be noted that the RP and NWE were excluded from the analysis of WPSH changes in daily data. The RP index is designed to measure the closed WPSH boundary, whereas on the synoptic scale, the western boundary of WPSH appears irregular or unclosed due to terrain influences. And the calculation of NWE requires daily meridional wind data, which are unavailable for most models at this time.

**Disagreements in WPSH**

In previous studies, inconsistencies in WPSH trends were exhibited across different WPSH definitions and datasets. We begin with the former disagreement by reproducing the trends of the WPSH between two previously proposed WPSH definitions, S-eddy and S-stmf. The two WPSH definitions, defined based on the geopotential height and stream function at single pressure level, represent thermodynamic- and dynamic-based metrics, respectively. NCEP1 shows (in blue line) a decreased Area Index (AI) in S-eddy during the past decades, implying the weakening of WPSH, whereas there is no significant change of AI in S-stmf (Fig. 1a). The changes in Intensity Index (II) and western Ridge Point index (RP) agreed with AI (see Fig. 1c, Fig. S1). This suggests that previously reported WPSH trends are sensitive to the choice of definitions^3,5^. Interestingly, there also are uncertainties in the WPSH trends across different datasets under the same definition, i.e., the second type of disagreement. For example, there is no trend of II in S-eddy for ERA5, unlike the decrease in NCEP1 and JRA55 (Fig. 1b); JRA55 shows a significant eastward retreat in S-stmf, whereas RP remains unchanged in ERA5 and NCEP1 (Fig. S1). Such a dataset dependence should not be simply attributed to discrepancies in the data themselves. It may be related to the different sensitivities of various WPSH definitions to data errors. Since different definitions employ different physical quantities (anomalies, vorticity) and exhibit inconsistent performance in capturing thermodynamic and dynamic features, which may amplify the errors among datasets and manifest as biases in the WPSH.

The spatial consistency between the WPSH and relevant weather anomalies also varies under different definitions. Currently, the S-5875 is the most used definition in operational diagnosis and prediction. The popularity of S-5875 is attributed to its ability to precisely outline the northwestern flank area of the WPSH, which area spans across rainband from southern China to Japan (black line in Fig. 1c). Furthermore, the interior of the WPSH, as delineated by S-5875 aligns well with clear skies and less precipitation resulting from descending airflow, which is invaluable for pinpointing the locations of abnormal temperatures and precipitation^9,11–13^. However, the boundaries of the WPSH defined by S-stmf or S-eddy (blue and gray line in Fig. 1c), notably cut cross the rainband. This poses challenges when using the WPSH boundary to diagnose the weather anomalies.

S-eddy and S-stmf focus on either atmospheric thermodynamic or dynamical aspects to define the WPSH, respectively. The differences in the responses of thermodynamic and dynamical aspects to warming may be the reason for the discrepancies in the long-term change and spatial coverage in different WPSH metrics. This is manifested in the fact that the high-pressure coverage of the WPSH has expanded over the past few decades, while the corresponding circulation features have not changed obviously^14,15^. However, the formation and maintenance of WPSH is associated with both dynamic forcing and thermodynamic adaptation processes. Specifically, the dynamic descent and accumulation of the northern branch of the Hadley cell ^16,17^, along with the enhanced latent heat released from rainfall during the monsoon season ^18–21^, are key drivers of the WPSH. While the weakening of the Hadley Circulation contrasts with the intensification of latent heat release over East Asia in the context of warming^22^, which may introduce uncertainties in the observed changes of the WPSH under differently focused definitional metrics. Therefore, it could be more appropriate to measure WPSH based on a thermodynamics-dynamics synergistic metric.

**Joint Probability Distribution Frequency (JPDF)**

**The definition of JPDF**

Since the formation and maintenance of the WPSH are attributed to the combined effects of atmospheric dynamics and thermodynamic processes, it is a more comprehensive way to measure the WPSH by considering both the thermodynamic and dynamical metrics. To achieve the above purpose, we define the WPSH based on the joint relationship between 500-hPa Z and ω.

The use of Z and ω is physically motivated. At a fixed pressure level, geopotential height is closely related to the column-mean temperature through the hypsometric relation,

$$\text{Z}_{\text{2}}\text{=}\text{Z}_{\text{1}}\text{+}\frac{\text{R}_{\text{d}}}{\text{g}}\int_{\text{p2}}^{\text{p1}} \text{T}_{\text{v}}\text{d}\ln\text{p}$$

where T_v_ is virtual temperature. Thus, 500-hPa Z reflects the thermodynamic state of the tropospheric column. Meanwhile, ω is dynamically linked to horizontal divergence and convergence through the continuity equation in pressure coordinates,

$$\frac{\text{∂}\text{ω}}{\text{∂p}}\text{=-}\text{∇}_{\text{h}}\text{∙}\vec{\text{V}}$$

where $\vec{V}$ is the horizontal wind. Therefore, ω provides a direct diagnostic of the vertical motion associated with the WPSH circulation. In this study, Z and ω are used together to identify a physically meaningful WPSH boundary that separates the relatively subsiding, hot, and dry region from the ascending and rainy region along the northwestern flank and relatively ascent of the WPSH.

The 500 hPa level is focused on for several reasons. First, the 500-hPa Z has been widely used in previous studies because it effectively captures the large-scale circulation characteristics and its weather impact. Therefore, using 500 hPa facilitates comparison with previous literature. Second, compared with lower-tropospheric levels such as 700, 850 hPa, the 500 hPa field is less affected by boundary-layer processes, land-sea thermal contrast, and topographic effects. Thus, it provides a relatively stable representation of the large-scale WPSH. Third, the 500-hPa level is closely related to mid-tropospheric subsidence and large-scale vertical motion.

We utilize the JPDF, which can measure the nonlinear relationships among meteorological variables. It is commonly used to analyze the synergistic changes among multiple variables in a changing climate. For instance, the JPDF can help us to understand the coordinated variability between the rising temperature of the Indo-Pacific Warm Pool and local deep convection under climate change ^23,24^. It is also used to analyze the relationship between the duration of extreme heatwaves and the precipitation intensity changes^13,15^. Therefore, the JPDF is a viable solution for measuring the WPSH through a thermodynamic-dynamic combined strategy.

The impact of WPSH is closely tied to its vertical motion. Assuming that the vertical motion of the WPSH remains constant, there will not be evident changes in its intensity. Therefore, exploring the variation in the Z coverage for certain value of ω can help understand changes in the WPSH. Technically, the Z-ω JPDF gives the area-weighted joint probability distribution frequency of certain values of ω and Z, which can be used to measure the relationship between Z and ω. The detailed procedure for deriving a Z-ω JPDF is as follows:

(1) Extract monthly observed or model-simulated ω and Z data inside the East Asia (10°N–40°N, 90°E–180°E);

(2) For each month, construct an area-weighted joint probability distribution (JPDF) by calculating the area of grid points with ω and Z values within the range of each bin.

$$\text{JPDF}\left( \text{Z}_{\text{i}}\text{,}\text{ω}_{\text{j}} \right)\text{=}\sum_{\text{k}} \text{A}_{\text{k}}\text{∙I[}\text{Z}_{\text{i}}\text{≤}\text{Z}_{\text{k}}\text{<}\text{Z}_{\text{i}}\text{+∆Z,}\text{ω}_{\text{j}}\text{≤}\text{ω}_{\text{k}}\text{<}\text{ω}_{\text{j}}\text{+∆ω]}$$

where A_k_ is the area of grid cell k, and I[$\cdot$] is an indicator function. I[$\cdot$] equals 1 when the grid point satisfies the condition, and 0 otherwise. $\Delta$Z and $\Delta\omega$ are the bin widths of JPDF, and are set 1.0 gpm and 0.002 Pa$\cdot$s^-1^, respectively. Thus, the JPDF represents the total area of grid points where Z and $\text{ω}$ fall into a given bin. The unit of JPDF is presented as km^2^;

(3) Derive the summer mean Z-ω JPDF by averaging the monthly Z-ω JPDF from June to August of each year;

(4) Plot the estimated frequency value in the form of a two-dimensional histogram, where the x-axis and y-axis indicate the Z and ω values, respectively (Fig. 2a and Fig. S2).

The calculation of the Z-ω joint cumulative distribution frequency (JCDF) is the same as that of the Z-ω JPDF, except for step (2). The frequency of each JCDF is estimated by calculating the area of grid points that are observed below each Z bin within a certain ω value.

$$\text{JCDF}\left( \text{Z}_{\text{i}}\text{,}\text{ω}_{\text{j}} \right)\text{=}\frac{\sum_{\text{k}} \text{A}_{\text{k}}\text{I[}{\text{Z}_{\text{l}}\text{≤}\text{Z}_{\text{i}}\text{,ω}}_{\text{j}}\text{≤}\text{ω}_{\text{k}}\text{<}\text{ω}_{\text{j}}\text{+∆ω]}}{\sum_{\text{k}} \text{A}_{\text{k}}\text{I[}\text{ω}_{\text{j}}\text{≤}\text{ω}_{\text{k}}\text{<}\text{ω}_{\text{j}}\text{+∆ω]}}\text{×100\%}$$

Thus, the Z-ω JCDF means that ω is fixed within a given bin, and only Z is accumulated. In other words, the JCDF quantifies the conditional area proportion of Z<=Z_i_ under a specified vertical-motion regime, thus the unit of JCDF is presented as %.

Here we use the JCDF value at 5875 gpm (Z_T_) in present climate as a reference to assess the relative change in Z-ω JPDF due to global warming. Specifically, the Z_T_ exhibits a closed boundary pattern in the climatological Z and has therefore been widely used to distinguish between the upward and downward motions associated with the WPSH^25,26^. Based on this, we define a benchmark vertical velocity (ω_T_) as the area-weighted mean ω of all grid cells whose Z values fall within the Z_T_ bin. Derived from the JPDF of the ERA5 dataset, ω_T_=−0.0147 Pa/s (corresponding to the intersection of the mean ω and Z_T_ in Fig. 2a) serves as a diagnostic metric to measure the vertical motion direction of the WPSH in the present study. It should be noted that the ω_T_ is not a proxy of upward/downward motion in the mathematical sense (i.e., ω=0 Pa/s), but rather a physical threshold of the WPSH boundary that distinguishes between upward airflows associated with more rainfall and downward airflows leading to less rainfall. In the NCEP/NCAR and JRA55 reanalysis, ω_T_ is -0.0202 Pa/s and -0.0134 Pa/s (Fig. S2c, S2d), respectively.

To estimate the boundary of WPSH, denoted as σ_JPDF_, we first derive the climatological cumulative probability (denoted as JCDF_T_) at the (Z_T_,ω_T_) from the climatic JCDF.

$$\text{JCDF}_{\text{T}}\text{=JCDF(}\text{Z}_{\text{T}}\text{,}\text{ω}_{\text{T}}\text{)}$$

In ERA5, the JCDF_T_ is 68.3%. This threshold implies that, for all grid points with ω_T_, the upper 31.7% of the Z distribution exceeds Z_T_, corresponding to WPSH-like characteristics. This JCDF_T_ is subsequently used as the JCDF threshold of Z-ω to estimate the annual σ_JPDF_. Specifically, for each year, we fix ω=ω_T_ and search for the Z value at which the seasonal-mean JCDF equals 68.3%. This Z value is defined as the annual JPDF-based WPSH boundary threshold σ_JPDF_.

$$\text{JCDF}_{\text{y}}\left( \text{σ}_{\text{JPDF}}\text{,}\text{ω}_{\text{T}} \right)\text{=}\text{JCDF}_{\text{T}}$$

For instance, in 1961, the value of Z satisfying JCDF_1961_(Z, ω_T_) =68.3% is 5864 gpm (Fig. S4c). This means that, among grid cells within the ω_T_ bin in 1961, 68.3% of the area has Z<=5864 gpm. Therefore, 5864 gpm is defined as σ_JPDF_ for that year. Similarly, in 2023, the same JCDF_T_=68.3% at ω_T_ corresponds to σ_JPDF_ =5892 gpm (Fig. S4d). By this way, σ_JPDF_ for each year can be calculated as shown in Fig 3b. For NCEP/NCAR and JRA55 reanalysis, the corresponding climatic JCDF_T_ values at (Z_T_,ω_T_) are 70.7% and 71.3%, respectively (Fig. S4a, 4b).

As the air column warms, σ_JPDF_ obviously increases with increasing temperature (Fig 3b). The growth rate of σ_JPDF_ in ERA5 and JRA55 is 3.55 and 3.30 gpm/decade, but that in NCEP/NCAR is larger (4.10 gpm/decade), which indicates that there are differences in the sensitivity of Z to global warming among datasets.

With the specification of σ_JPDF_, the S-JPDF method provides an objective boundary for identifying the WPSH. Based on this boundary, WPSH indices can be calculated and compared with those obtained from previous definitions. To facilitate understanding of the full procedure, we also provide a schematic overview of the workflow for the JPDF-based definition of the WPSH in Fig. S11. In the following sections, we give the interpretation, sensitivity, and validation of the S-JPDF method.

**Sensitivity of (Z_T_, ω_T_) relationship**

The derived ω_T_ partly depends on the selected reference Z_T_, and one could estimate it based on other physically reasonable Z_T_. We tested the sensitivity of ω_T_ by deriving it from a range of reference Z_T_. As shown by the black line in Fig. 3a, Z increases nonlinearly with increasing ω, while the slope of the curve (thin black line) exhibits a consistently linear increase. Over the range of ω=-0.04 to 0 Pa/s, the slope of the curve is about 0.47 gpm/(Pa/s). This indicates that the Z_T_-ω_T_ relationship is well constrained in current climate.

We further checked the sensitivity of Z_T_-ω_T_ relationship under different climate states. From Fig. 3a, the Z against ω in different past climate shifts upward with time (colored thick lines), mainly due to the overall increase in Z. However, the slope of the curve (colored thin lines) remains similar across different periods. Moreover, the same diagnostic was also performed for the future projections. The slope of the curve remains highly consistent across different SSP scenarios and future periods (Fig. 5h, S9e, 5i, S9f).

The nearly unchanged slope of ω-Z curve under different climate backgrounds suggests that the coupling between Z and ω near the WPSH boundary remains relatively stable. In other words, warming mainly produces an overall upward shift of the Z field through thermal expansion of the air column, rather than fundamentally changing the dynamical meaning of a given vertical motion state. Therefore, the vertical motion reference ω_T_, can still be regarded as a robust indicator of the WPSH boundary condition in warmer climates. This provides support for using the JCDF-based threshold σ_JPDF_ to define a time-varying WPSH boundary.

**Classification of Physical Regimes in JPDF**

To further clarify the mechanisms in characterizing the WPSH by JPDF, we project the JPDF phase separated by (Z_T_, ω_T_) and onto the spatial map (Fig. S3). The 1st quadrant represents the case of more subsiding (ω > ω_T_) with a higher Z (Z > Z_T_) regime, whose spatial distribution of frequency is similar to the traditional perception of the WPSH pattern. The 2nd quadrant shows the case of more subsiding (ω>ω_T_) with a lower Z (Z<Z_T_) regime, which mainly occurs out of subtropical regions. The 3rd quadrant represents the frequency of more ascending (ω<ω_T_) with lower Z (Z< Z_T_), which corresponds to the upward airflow over landmass and tropical regions. And the fourth quadrant gives the case of more ascending (ω<ω_T_) with higher Z (Z> Z_T_), which rare occurred. Thus, the JPDF essentially categorizes the atmospheric states based on the synergistic distribution between the ω and Z.

**Validation of S-JPDF**

To verify the performances of WPSH in S-JPDF in diagnosing weather and climate anomalies, as well as its advantages over previous definitions, we conduct composite analyses of extreme precipitation at both climatic and synoptic scales, respectively. Fig. 4a-4b gives the composite results for the top 5 wettest and 5 driest summers over Sichuan-Yangzi Basin of China (26°N-33°N, 104°E-121°E) from 1961-2023. It is found that the WPSH boundary in S-JPDF and S-5875 match well with the rainbands and upward airflow zone on its northwest side, but S-eddy and S-stmf do not (Fig. S5). Early studies using S-5875 have proved that the WPSH is highly correlated with precipitation anomalies in eastern China, with stronger WPSH generally corresponding to wet summer and vice versa ^27,28^. The index differences in S-JPDF between dry and wet cases exhibits similar results ($\Delta$AI>0, $\Delta$II>0, and $\Delta$RP<0, see Table S1), which is more evident than those obtained from S-stmf and S-eddy. This implies that the WPSH defined by S-JPDF corresponds well with precipitation anomalies. Moreover, we selected the 16 strongest precipitation cases in 1961-2023 based on anomalies of Px5day (5-day running-mean precipitation), defined as the Px5day exceeding 1.96 standard deviations. In all 16 cases (Fig. S6), the northwest side of the WPSH boundary in S-JPDF well matches updrafts and precipitation zones. However, in some cases of S-5875 (case 1964, 1980, 1991, and 1993, black line) and S-eddy (1980, 1991, 1993 and 1998, grey line), this relationship is not well captured. Moreover, the WPSH boundary in S-stmf crossed the precipitation zone in most cases, failing to capture the physical connection between the WPSH and the precipitation area. Based on the mean of 16 cases (Fig. 4c), the WPSH boundary and the precipitation zone are well coupled in S-JPDF, which implies that the WPSH in S-JPDF performed well in the diagnosis of extreme precipitation zones.

A scorching summer is always accompanied by a strong WPSH^2,7^. Next, we conducted a similar composite analysis on the temperature anomalies. The WPSH boundary in S-JPDF matches well with the downward airflow that favors high temperatures (Fig. 4d-4f, S5). And the index differences in S-JPDF illustrate a stronger WPSH during a hotter summer ($\Delta$AI>0, $\Delta$II>0, and $\Delta$RP<0, Table S1). In contrast, in S-eddy and S-5875, the differences in AI and II do not support the same result, despite giving a more westward RP in hotter summers. The WPSH index differences in S-stmf are also less pronounced than those defined by S-JPDF. In the hottest 16 cases selected based on Tx5day (5-day running-mean 2m temperature) anomalies, the downward airflow inside the WPSH boundary in S-JPDF matches well with the land hot area (Fig. S5), while the remaining three WPSH definitions seem to perform less well. For example, in S-5875, no WPSH boundary is identified in some cases despite the occurrence of downward motion over the hot region, such as in 1961, 1988, and 2013. Similar issues appeared in S-eddy (case 1964, 1988, 2006, and 2013) or S-stmf (case 1961, 1964, 1988, 2003, 2006, 2013, and 2017). When considering the mean of the 16 cases, the WPSH coverage defined by S-JPDF is consistent with both the downward-motion region and the hot area comparing with the other definitions, further demonstrating the advantage of the S-JPDF method in diagnosing heat-related WPSH impacts.

**Linear scaling correction**

Given that the CMIP6 is capable of reproducing the patterns of Z-ω JPDF, there exist biases only in the climatology of Z values. Thus, we employed a linear scaling correction method to correct the climatological bias of Z. The linear scaling correction aims to perfectly match the monthly mean of corrected values with that of observed ones and keeps the raw variability ^29^. It operates with monthly or daily correction values based on the differences between observed and raw model outputs. An additive term will be added to the values of the variable (Z in this case).

Z_cor,m_=Z_raw,m_+µ(Z_obs,m_)- µ(Z_raw,m_)

where Z_cor,m_ and T_raw,m_ are corrected Z and Z raw model output on the m month, µ() represents the multi-year average of Z for given month m.

Figure S1 Temporal evolution of the summer WPSH indices measured by (a-c) the S-eddy and (d-f) the S-stmf strategies. The AI, II, and RP indices are presented in separate panels. The red, blue, and gray lines represent results from the ERA5, NCEP1, and JRA55 reanalysis datasets, respectively. The values above each panel indicate the trend (per decade). Due to the different units of the II from the two definition strategies, standardized values are used for comparison (the trend and significance are consistent before and after standardization). The asterisk (*) denotes the trend is statistically significant at the 99% confidence level.

Figure S2 Z-ω JPDF over Eastern Asia. (a,b) show the distribution from ERA5 at 500hPa for the periods 1961-1980 and 2002-2024, respectively. (c,d) show the distribution from NCEP1 and JRA55 at 500 hPa for the periods 1961-2023, respectively. (e, f) show the distribution from ERA5 at 700 hPa and 850 hPa for the periods 1961-2023, respectively. The superimposed gray curve denotes the mean ω value for each 1-gpm Z bin, averaged over all grid points at the corresponding pressure level.

Figure S3 Spatial distribution of Z-ω frequency for different JPDF quadrants. (a) Quadrant 1: ω >= ω_T_, Z >= Z_T_; (b) Quadrant 2: ω >= ω_T_, Z < Z_T_; (c) Quadrant 3: ω < ω_T_, Z < Z_T_; (d) Quadrant 4: ω < ω_T_, Z >= Z_T_.

Figure S4 (a) Z-ω JCDF over Eastern Asia over 1961-2023 in (a) NCEP1 and (b) JRA55 datasets, and in (c) 1961 and (d) 2023 in the ERA5 dataset. Thick black solid curves represent the 70.7%, 71.3%, 68.3% contour lines of the JCDF_T_ in NCEP1, JRA55, and ERA5, respectively, smoothed using a 9-point running mean filter.

Figure S5 Distribution of precipitation for (a-e) the 5 wettest, (f-j) the 5 dryest summers, and distribution of land 2m air temperature anomalies for (a-e) the 5 hottest, (g-k) the 5 coldest summers in the ERA5 dataset, as well as the WPSH boundary based on different definitions. The black, red, gray, and blue solid lines represent the WPSH boundary in S-5875, S-JPDF, S-eddy, and S-stmf, respectively.

Figure S6 (a-f) The spatial distribution of Px5day anomalies during the top 16 extreme precipitation events from 1961 to 2023, along with the WPSH boundaries defined by different strategies (red, black, gray, and blue represent the results of S-JPDF, S-5875, S-eddy and S-stmf, respectively). Dotted shading is where upward airflow occurs (ω < -0.0147). (q) Same as (a-f) but for the average of all events. (r) The evolution of the 16 extreme precipitation events, with the thin solid black line representing the Px5day in 1998 (the top 1 among 16 events). The thick solid black line is the Px5day anomaly relative to the 1981-2010 seasonal cycle across 1961-2023. The dashed black line and gray shading represent the range of Px5day (90% and 99% confidence intervals of the interannual variability). The top 16 events are selected based on the 99% confidence interval, and their duration is depicted by colored solid lines.

Figure S7 (a-f) The spatial distribution of Tx5day anomalies during the top 16 extreme heat events from 1961 to 2023, along with the WPSH boundaries defined by different strategies (red, black, gray, and blue represent the results of S-JPDF, S-5875, S-eddy and S-stmf, respectively). Dotted shading is where downward airflow occurs (ω >= -0.0147). (q) Same as (a-f) but for the average of all events. (r) The evolution of the 16 extreme heat events, with the thin solid black line representing the Tx5day in 2022 (the top 1 among 16 events). The thick solid black line is the Tx5day anomaly relative to the 1981-2010 seasonal cycle across 1961-2023. The dashed black line and gray shading represent the range of Tx5day (90% and 99% confidence intervals of the interannual variability). The top 16 events are selected based on the 99% confidence interval, and their duration is depicted by colored solid lines.

Figure S8 (a-d) Temporal evolution of the II (units: 10^5^km^2^·gpm), RP (units: °E), NWE (units: -) and LandAI (units: 10^5^km^2^) indices of WPSH defined by S-JPDF. The red, blue, and gray lines represent results from ERA5, NCEP1, and JRA55 datasets. The trend of each reanalysis is shown above the panel (per decade), and all trends are insignificant at the 99% confidence level.

Figure S9: (a) The box-and-whisker plot of the uncertainty of average Z and ω over East Asia in 26 CMIP6 models spanning the period 1961-2023, with gray shading representing the uncertainty of reanalysis datasets; (b) Same as Fig. 2(a) but for the MME of CMIP6 instead, the gray solid line and shading show the averaged ω corresponding to different Z bins for the MME and individual models, and the black contours show the trend (decade^-1^, only trends being significant at the 95% confidence level are plotted), with negative trends for dashed lines and positive trends for solid lines; (c) Same as Fig. S3(a) but for MME of CMIP6, which represents the spatial distribution of the frequency where ω >= ω_T_ and Z >= Z_T_; (d) Same as Fig. 2(c) but for the MME, with dashed lines showing the negative trend (units: decade^-1^, only trends significant at the 95% confidence level are plotted). Thick black solid curves display the JCDF_T_(67.2%) of the Z-ω JCDF. The grey contour represents the Z-ω JPDF at a value of 0.01 10^7^*km^2^. (e, f) Same as Fig. 5 (h, i) but for the curves of Z against ω under SSP1-2.6 and SSP3-7.0 scenarios.

Figure S10 Temporal evolution of simulated a) AI (units: 10^5^km^2^), and b) NWE (units: -) indices of WPSH in MME over 2015-2100. The trend of each scenario is shown above the panel (per decade). The PDF of c) AI, d) RP and e) NWE anomalies for the historical (1965-2014) and SSPs (2051-2100) run. The digits in the fig. (c-e) represent the average (Avg), standard deviation Dev) of the corresponding period. The grey, blue, green and red lines represent the results of SSP1-2.6, SSP2-4.5, SSP3-7.0, and SSP5-8.5.

Figure S11 Overview of the workflow for the JPDF-based definition of the WPSH.

Table S1 Composite difference in AI (units: 10^5^km^2^), II (units: 10^5^km^2^·gpm) and RP (units: °E) Indices between the top 5 high and the top 5 low summers for precipitation and temperature anomalies.

|  | Precipitation | | | Temperature | | |
| --- | --- | --- | --- | --- | --- | --- |
|  | $\Delta$AI | $\Delta$II | $\Delta$RP | $\Delta$AI | $\Delta$II | $\Delta$RP |
| S-5875 | 31.99 | 40.88 | -17.00 | -2.12 | -13.02 | -1.25 |
| S-eddy | 1.81 | 3.82 | -5.25 | 3.26 | -2.56 | -0.50 |
| S-stmf | 7.62 | 0.06 | -7.25 | 8.49 | 0.01 | -3.50 |
| S-JPDF | 27.07 | 36.83 | -15.75 | 22.55 | 23.19 | -9.25 |

Table S2 Details of 26 models used in this study. Models marked with * were used for the synoptic scale WPSH analysis.

| Models | Resolution | Models | Resolution |
| --- | --- | --- | --- |
| ACCESS-CM2* | 1.25 ×1.875 | FGOALS-f3-L | 1 ×1.25 |
| ACCESS-ESM1-5 | 1.25 ×1.875 | FGOALS-g3* | 2.0x2.0 |
| AWI-CM-1-1-MR | 0.94 ×0.94 | GFDL-ESM4 | 1 ×1.25 |
| BCC-CSM2-MR | 1.125 ×1.125 | INM-CM4-8* | 1.5 ×2 |
| CanESM5* | 2.813 ×2.813 | INM-CM5-0* | 1.5 ×2 |
| CanESM5-1 | 2.813 ×2.813 | IPSL-CM6A-LR* | 1.26 ×2.5 |
| CAS-ESM2-0 | 1.4 ×1.4 | MIROC6* | 1.4 ×1.4 |
| CESM2-WACCM* | 0.94 ×1.25 | MPI-ESM1-2-HR* | 0.94 ×0.94 |
| CMCC-CM2-SR5 | 0.94 ×1.25 | MPI-ESM1-2-LR* | 1.875 ×1.875 |
| CMCC-ESM2 | 1.875 ×1.875 | MRI-ESM2-0* | 1.125 ×1.125 |
| EC-Earth3* | 0.7 ×0.7 | NorESM2-LM* | 1.9 ×2.5 |
| EC-Earth3-Veg | 0.7 ×0.7 | NorESM2-MM* | 0.94 ×1.25 |
| EC-Earth3-Veg-LR | 1.125 ×1.125 | TaiESM1* | 0.94 ×1.25 |

Table S3 Abbreviations used in the context.

| Abbreviation | Full name |
| --- | --- |
| WPSH | Western Pacific Subtropical High |
| Z | Geopotential height |
| ω | Vertical velocity |
| JPDF | Joint Probability Distribution Frequency |
| JCDF | Joint Cumulative Distribution Frequency |
| S-JPDF | WPSH definition based on the JPDF method |
| S-5875 | WPSH definition based on the 5875-gpm geopotential-height threshold |
| S-eddy | WPSH definition based on eddy geopotential height |
| S-stmf | WPSH definition based on streamfunction |
| AI | Area index |
| II | Intensity index |
| RP | Western Ridge Point index |
| RL | Westward Extending Ridge Line index |
| NWE | Northwest Extension index |
| LandAI | Land Area index |
| Px5day | 5-day running-mean precipitation |
| Tx5day | 5-day running-mean 2m temperature |

**References for Supplementary Data:**

1.	Li Z, Ren H, Lu M *et al*. Interannual variations of westward extension area of western Pacific subtropical high and its relationship with precipitation in East Asia. *Atmospheric Res* 2024; 298: 107148.

2.	Li N, Xiao Z, Zhao L. A Recent Increase in Long-Lived Heatwaves in China Under the Joint Influence of South Asia and Western North Pacific Subtropical Highs. *J Clim* 2021; **34**: 7167–7179.

3.	Lu R. Indices of the Summertime Western North Pacific Subtropical High. *Adv Atmospheric Sci* 2002; **19**: 1004–1028.

4.	Jia B, Peng J, Hu S *et al*. Novel dynamical indices for the variations of the western Pacific subtropical high based on three-pattern decomposition of global atmospheric circulation in a warming climate. *Clim Dyn* 2021; **56**: 3473–3487.

5.	Li D, Hu S, Wu Y *et al*. Which metric of the WNPSH index can be better applied for practical applications. *Clim Dyn* 2025; **63**: 319.

6.	Huang Y, Li X, Wang H. Will the western Pacific subtropical high constantly intensify in the future? *Clim Dyn* 2016; **47**: 567–577.

7.	Zhou F, Siseho S-S, Liu M et al. Joint Impacts of the North African and the Western Pacific Subtropical Highs on Summer Precipitation over the Tibetan Plateau. J Clim 2024; 37: 3825–3846.

8.	Choi W, Kim K-Y. Summertime variability of the western North Pacific subtropical high and its synoptic influences on the East Asian weather. Sci Rep 2019; 9: 7865.

9.	He C, Zhou T, Zhang L *et al*. Extremely hot East Asia and flooding western South Asia in the summer of 2022 tied to reversed flow over Tibetan Plateau. *Clim Dyn* 2023; **61**: 2103–2119.

10.	Chen R, Li X. Causes of the persistent merging of the western North Pacific subtropical high and the Iran high during late July 2022. *Clim Dyn* 2023; **61**: 2285–2297.

11.	Yuan Y, Gao H, Ding T. The extremely north position of the western Pacific subtropical high in summer of 2018: Important role of the convective activities in the western Pacific. *Int J Climatol* 2020; **40**: 1361–1374.

12.	Li N, Xiao Z, Zhao L. A Recent Increase in Long-Lived Heatwaves in China Under the Joint Influence of South Asia and Western North Pacific Subtropical Highs. *J Clim* 2021; **34**: 7167–7179.

13.	Duan R, Huang G, Zhou X *et al*. Record-Breaking heavy rainfall around Henan Province in 2021 and future projection of extreme conditions under climate change. J Hydrol 2023; **625**: 130102.

14.	He C, Lin A, Gu D *et al*. Using eddy geopotential height to measure the western North Pacific subtropical high in a warming climate. *Theor Appl Climatol* 2018; **131**: 681–691.

15.	Zhu H, Jiang Z, Li L *et al*. Improve the projection of East China summer precipitation with emergent constraints. *Npj Clim Atmospheric Sci* 2024; **7**: 298.

16.	He J, Zhou B, Wen M *et al*. Vertical circulation structure, interannual variation features and variation mechanism of western pacific subtropical high. *Adv Atmos Sci* 2001; **18**: 497–510.

17.	Li W, Li L, Ting M *et al*. Intensification of Northern Hemisphere subtropical highs in a warming climate. *Nat Geosci* 2012; **5**: 830–834.

18.	Lu R. Indices of the Summertime Western North Pacific Subtropical High. *Adv Atmospheric Sci* 2002; **19**: 1004–1028.

19.	Liu W, Shi N, Wang H. Evolution of the western North Pacific subtropical high and impact of Asian precipitation from spring to summer. *Atmospheric Res* 2025; **315**: 107909.

20.	Liu Y, Wu G. Reviews on the study of the subtropical anticyclone and new insights on some fundamental problems. *J Meteorol Res* 2000; **58**: 500–512.

21.	Zhao T, Fu C. Intercomparison of the Summertime Subtropical High from the ERA-40 and over East Eurasia and the western North Pacific. *Adv Atmospheric Sci* 2009; **26**: 119–131.

22.	Tian Z, Hu D, Lang X *et al*. Index‐ and model‐dependent projections of East Asian summer monsoon in Coupled Model Intercomparison Project Phase 6 simulations. *Int J Climatol* 2022; **42**: 2208–2224.

23.	Kim H-R, Ha K-J, Lau W. Changes in the SST-precipitation relationship over the Indo-Pacific warm pool under a warming climate. *Environ Res Lett* 2023; **18**: 074031.

24.	Leung J, Zhang B, Gan Q *et al*. Differential expansion speeds of Indo-Pacific warm pool and deep convection favoring pool under greenhouse warming. *Npj Clim Atmospheric Sci* 2022; **5**: 97.

25.	Yuan Y, Gao H, Ding T. The extremely north position of the western Pacific subtropical high in summer of 2018: Important role of the convective activities in the western Pacific. *Int J Climatol* 2020; **40**: 1361–1374.

26.	Duan R, Huang G, Zhou X *et al*. Record-Breaking heavy rainfall around Henan Province in 2021 and future projection of extreme conditions under climate change. J Hydrol 2023; **625**: 130102.

27.	Li Z, Ren H, Lu M *et al*. Interannual variations of westward extension area of western Pacific subtropical high and its relationship with precipitation in East Asia. *Atmospheric Res* 2024; 298: 107148.

28.	Wang B, Xiang B, Lee J-Y. Subtropical High predictability establishes a promising way for monsoon and tropical storm predictions. *Proc Natl Acad Sci* 2013; **110**: 2718–2722.

29.	Fang G, Yang J, Chen Y *et al*. Comparing bias correction methods in downscaling meteorological variables for a hydrologic impact study in an arid area in China. *Hydrol Earth Syst Sci* 2015; **19**: 2547–2559.
